# A framework for preferred practices in conducting culturally competent health research in a multicultural society

**DOI:** 10.1186/s12961-020-00657-y

**Published:** 2021-02-18

**Authors:** Lisa Woodland, Ilse Blignault, Cathy O’Callaghan, Ben Harris-Roxas

**Affiliations:** 1 Priority Populations, Population and Community Health, South Eastern Sydney Local Health District, 301 Forbes St, Darlinghurst, NSW 2010 Australia; 2 Translational Health Research Institute, School of Medicine, Western Sydney University, Translational Health Research Institute, Building 3, David Pilgrim Avenue, Campbelltown, NSW 2560 Australia; 3 South Eastern Sydney Research Collaboration Hub, Centre for Primary Health Care and Equity, University of NSW, Level 3, AGSM Building, UNSW, Sydney, NSW 2052 Australia; 4 Population and Community Health, South Eastern Sydney Local Health District, 301 Forbes St, Darlinghurst NSW 2010, Sydney, NSW 2010 Australia

**Keywords:** Multicultural, Research, Ethnic minorities, Culturally and linguistically diverse, Cultural competence, Community-based research

## Abstract

**Background:**

Improving the health and well-being of the whole population requires that health inequities be addressed. In an era of unprecedented international migration, meeting the health care needs of growing multicultural or multiethnic societies presents major challenges for health care systems and for health researchers. Considerable literature exists on the methodological and ethical difficulties of conducting research in a cross-cultural context; however, there is a need for a framework to guide health research in multicultural societies.

**Methods:**

The framework was informed by “research on research” that we have undertaken in community and primary health care settings in Sydney, Australia. Case studies are presented as illustrative examples.

**Results:**

We present a framework for preferred practices in conducting health research that is culturally informed, high-quality, safe, and actionable.

**Conclusions:**

The framework is not intended to be universal, however many of its aspects will have relevance for health research generally. Application of the framework for preferred practices could potentially make health research more culturally competent, thus enabling enhanced policies, programmes and practices to better meet population health needs. The framework needs to be further tested and refined in different contexts.

## Background

Modern Australia, established on the lands of Aboriginal and Torres Strait Islander peoples, is an “immigration nation” and one which is becoming increasingly culturally diverse [1]. Through successive waves of migration, particularly since the Second World War and the end of the White Australia Policy [2], this former British colony has evolved into a nation of over 25 million people from over 190 different countries and 300 different ancestries, with over 300 separately identified languages spoken at home [3].

Australia’s health system is considered one of the best in the world [4]. It is supported by a broad national programme of health and medical research that includes biomedical science, clinical medicine and science, public health and health services [5]. Meeting the health care needs of a growing multicultural society presents a major challenge for the health system and, therefore, for health researchers.

In this article we present a framework for preferred practices to guide health research that is culturally informed, high-quality, safe, and actionable; illustrating it with examples from our own work in Sydney, Australia’s largest and most multicultural capital city. Research in a multicultural context may be inclusive (research which does not systematically exclude people from culturally and linguistically diverse (CALD) backgrounds, especially those with low English proficiency) or targeted (research which specifically targets people from CALD backgrounds and their communities)—see Box 1 for additional definitions. Our focus here is on targeted research in community and primary health care settings. We suggest that successful research is conducted by a culturally competent research team that addresses systemic health inequities and community needs, being mindful of the power differentials involved. Challenges to incorporating these elements as part of routine practice are discussed.

Box 1: Definition of terms We use the following terms as they are commonly defined in Australian Government policy and health research:

Multicultural is a term that recognizes Australia’s culturally diverse population; is based on the shared values of respect, equality and freedom; and recognizes the need for shared rights and responsibilities [1].

Culturally and linguistically diverse (CALD) refers to “the non-Indigenous cultural and linguistic groups represented in the Australian population who identify as having cultural or linguistic connections with their place of birth, ancestry or ethnic origin, religion, preferred language or language spoken at home” (p. 3) [12].

Community is a group of people sharing common interests, perspectives, values and/or approaches but not necessarily a geographic association [29].

Consumers are “patients and potential patients, carers, and people who use health care services” (p. 6) [29].

### Multicultural landscape

As at the 2016 Census, 28% of Australia's population were born overseas, [3] a level that is higher than most countries within the Organisation for Economic Co-operation and Development (OECD) [6]. Another 21% of the population had one or both parents who were born overseas [3]. Permanent migrants enter Australia via one of two formal programmes, the Migration Program for skilled and family migrants, or the Humanitarian Program for refugees and those in refugee-like situations [6]. Recent years have seen increasing numbers of temporary migrants eligible to stay long-term (12 months or more) including students, temporary workers, and working-holiday makers.

Most migrants move to capital cities mirroring the global trend to urbanization [7]. In 2016, the population of Greater Sydney was 4,823,991, of whom 42.9% were overseas-born and 41.6% spoke a language other than English at home [8]. The most common overseas countries of birth were China 4.7%, England 3.1%, India 2.7%, New Zealand 1.8% and Vietnam 1.7% [8]. Languages other than English spoken at home included Mandarin 4.7%, Arabic 4.0%, Cantonese 2.9%, Vietnamese 2.1% and Greek 1.6% [8]. Almost one in five people (18.6.%) born overseas arrived between 2011 and 2016, and 14.6% reported they were not proficient in English [9].

### Health research landscape

Successfully addressing the needs of disadvantaged or marginalized groups (populations outside the mainstream society) will contribute to improved health for the whole population [10]. In Australia in recent years, most attention and an increasing amount of health research has properly focussed on Aboriginal and Torres Strait Islander peoples where the greatest health disparities exist [11]. CALD populations are a heterogeneous group and their health is affected by a range of factors including migration and settlement experiences, unfamiliarity with the health system, and level of English proficiency [12].

Inequities in the health care experiences of people with low English proficiency compared to the general population have been linked to increases in medical errors, hospital length of stay and readmissions [13–15]. Culture and language also affect how people understand and manage their health and access services, including experiences of racism and discrimination [16]. Certain country of birth groups have increased risk factors such as smoking, obesity, inadequate physical activity and increased rates of coronary heart disease and diabetes, compared to the general population [12]. The *NSW Health Plan for Healthy Culturally and Linguistically Diverse Communities 2019–23* recognizes the need to address these health inequities and outlines a vision for "an equitable, accessible and safe health system that ensures cultural and linguistic diversity is recognized and addressed in policy development, service planning and delivery” (p. 5) [12].

Accurate and meaningful data are essential to identify, understand and address health disparities. Both inclusive and targeted research are required. Traditionally, CALD populations have been under-represented in Australian health research [17, 18]. A review of Australian Research Council (ARC) and National Health and Medical Research Council (NHMRC) funded initiatives from 2002 to 2011 found only 7.8% of ARC people-focused projects and 6.2% of NHMRC people-focused projects were migrant-related [19]. A title scan of 500 NHMRC Project Grants from 2015 to 2019 undertaken by the authors revealed that less than 1% identified CALD populations as the project’s focus [20]. Having a preferred language other than English has been identified as a primary reason for the exclusion of people from CALD backgrounds in cancer research [21]. Australian health data sets, health data collections and population surveys contain several indicators of cultural and linguistic diversity [22]. In the state of New South Wales, “country of birth” and “main language spoken at home” have been the most commonly used variables in health research [23].

Cross-cultural research in multicultural and multilingual settings presents numerous methodological and ethical challenges [24–26]. Barriers to CALD consumer and community involvement include research that is not culturally appropriate, participatory or respectful of their needs [26]. These barriers have been well documented internationally [27]. Since 2006, the NHMRC has published three important resources in this area, the first being *Cultural Competency in Health: A guide for policy, partnerships and participation* [28]. The *Statement on Consumer and Community Involvement in Health and Medical Research*, co-authored by the NHMRC and the Consumers Health Forum of Australia, aims to guide research institutions, researchers, consumers and community members in the active involvement of consumers and community members in all aspects of health and medical research [29]. The *National Statement on Ethical Conduct in Human Research 2007 (Updated 2018)* makes several references to respect for cultural diversity and calls for researchers to reflect on the social and cultural implications of their work [30].

## Methods

The framework for preferred practices, which was informed by the authors’ collective experiences conducting multicultural health research over several decades, was developed and refined over three half-day meetings. Our academic qualifications include applied anthropology, social work, psychology, health promotion, applied social research and public health (see Authors’ information). Our professional experience includes roles in health service management, clinical services, multicultural services, hospitals and community health, developing and delivering cultural competence capacity building, public policy, health service research, social research, evaluation, and consulting.

The process for developing the framework was informed by interpretive description [32]. We had two aims for enhancing the validity of the framework development. The first was to ensure representative credibility, so that the data (case studies) were representative of the phenomenon described, that is, culturally competent health research in a multicultural society [32]. The second was interpretive authority (Altheide and Johnson 1994), so that we can be confident that the understandings described represented more widespread truths [33]. We addressed these aims through two one-day workshops between the authors, review of case studies and an iterative, collaborative drafting process.

The process began with us reflecting on successful culturally competent projects in which we had been involved, both together and separately. From these, a number were selected as case studies for further scrutiny at two one-day workshops, with consideration given to aims, methods, findings, and significance and impact. The case studies were then reviewed by the authors to identify common higher-order factors that enhance the conduct and impact of this type of research, based on an interpretative description approach [34]. Four of the projects are described in Tables 1, 2, 3 and 4.

**Table 1:** Example 1, *Fear and Shame*: Using theatre to destigmatize mental illness in an Australian Macedonian community

Overview of the research	How we conducted the research	Outcomes
Fear and Shame*:* Using theatre to destigmatize mental illness in an Australian Macedonian community [36.^27^] This study evaluated an innovative mental health promotion initiative in which applied theatre^a^ was used to promote mental health literacy and reduce stigma within the Macedonian community Qualitative data from earlier studies and professional experience of the bilingual mental health clinician and playwright were used to create the in-language play, which capitalized on the strong history of theatre in the Macedonian community. Eight performances at three venues in Sydney and Wollongong were attended by approximately 1600 people Funding source: South Eastern Sydney Local Health District	Recruitment and consent: Participants were audience members recruited either retrospectively through Macedonian community groups or at the venue after the performance. Trained bilingual interviewers contacted potential respondents by telephone and obtained verbal consent. Interviews were conducted in Macedonian or English according to respondent preference Data collection and analysis: Interviews with 236 audience members (including 76 with personal or family experience of mental illness) and 25 key informants (service providers and community leaders) were conducted 1–10 months after the play was performed. Respondents were asked about attitudes towards mental illness and help-seeking behaviour using the same questions asked in earlier research. Responses were recorded in either Macedonian or English and translated as necessary by the interviewer. Data were analysed and compared with data collected 6 years earlier	Findings: The play sparked a community conversation about mental illness and the key messages were well received. Over the period 2003–2009, the play contributed to more positive attitudes towards mental illness, a reduction in the stigma surrounding mental illness, and improved knowledge of mental health services. Respondents indicated greater willingness to seek help from health services and key informants reported greater service utilization after the play was staged Significance and impact: The study demonstrated the value of applied theatre as a vehicle for mental health promotion in the Macedonian community. Following the play’s success, *Fear and Shame* was translated and culturally adapted for the Greek community, and used as a case study in an e-learning tool to enhance cultural competency of mental health clinicians. The project was a finalist in the NSW Health Awards, Promoting Health Category, 2010

^a^“Applied theatre” refers to theatre used for educative and/or social change purposes

**Table 2:** Example 2, Longitudinal study of refugee children

Overview of the research	How we conducted the research	Outcomes
Longitudinal study of refugee children and their health, development and social-emotional well-being. [38–40] This prospective cohort study examined physical health, development, and social and emotional well-being of newly arrived refugee children (ages 6 months to 15 years) settling in a regional part of NSW Participants were recruited between 2009 and 2013 and followed up for approximately two and a half years post arrival. Participants came from 10 countries of origin (predominantly from World Health Organization (WHO) African and South-East Asia regions); representing 10 language groups (most commonly Burmese, Karen, Swahili and Arabic) Funding source: Financial Markets Foundation for Children and South Eastern Sydney Local Health District	Recruitment and consent: Sixty-one subjects were enrolled in the study at Year 1 through an existing model of care. Only two children per family were enrolled to minimize participant burden on families. Refugee Health Nurses (RHNs) (clinician researchers) enrolled and obtained consent from participants with the support of face-to-face professional health care interpreters (HCIs). Individual HCI were briefed and provided with orientation to the research instruments prior to the assessments being undertaken. There was 100% retention at Year 2 (average 13 months post arrival) and 85% retention at Year 3 (average 31 months post arrival) Data collection and analysis: General practitioners conducted early health assessments at Year 1 (as per the existing model of care). The research team conducted follow up assessments at Year 2 and Year 3 in the child’s home by the RHN with the support of a face-to-face HCI. Outcome measures included the play based/observational Australian Developmental Screening Test (ADST); and the parent-completed Strengths and Difficulties Questionnaire (SDQ). Translated and validated versions of the SDQ were used where available and appropriate. Structured parent interviews including items from the Social Readjustment Rating Scale (SRRS) assessed family and settlement risk and protective factors	Findings: This study demonstrated that a longitudinal cohort study in refugee children is feasible and acceptable, and retention rates can be high. Development and social-emotional well-being of resettled refugee children improved for the majority of children over 2 to 3 years. However, a minority had persistently poor social-emotional outcomes. The study identified a number of risk and protective factors over the first years of refugee settlement including post-arrival factors that are modifiable through policy and practice interventions Significance and impact: This study is one of the first of its kind to examine the prevalence of child, family and settlement factors that may impact on refugee children’s health, development and social-emotional well-being over the first years of refugee settlement. The study fills a gap in the evidence base for policy and practice development. The study influenced the NSW government response to Iraqi and Syrian refugees fleeing the Syrian crisis, with funding provided for specialized early childhood nursing services (a health service gap identified by the study)

**Table 3:** Example 3: Chinese Get Healthy Service (CGHS)

Overview of the research	How we conducted the research	Outcomes
Effectiveness of the NSW Get Healthy Information and Coaching Service among Chinese communities [41, 42] This qualitative study explored participant and stakeholder perceptions of the GHS with Chinese (Mandarin and Cantonese speaking) communities in Sydney. This complemented a broader quantitative study of the service The CGHS is a cultural adaptation of an existing GHS programme. It is a free telephone-based lifestyle programme, provided over a 6-month period by qualified bilingual/ bicultural coaches. The programme offers resources in Simplified and Traditional Chinese and is promoted through Chinese community organizations and networks Funding source: NSW Office of Preventative Health	Recruitment and consent: A trained bilingual research assistant (BRA) recruited Mandarin and Cantonese speaking GHS participants who had completed the programme through participant registration lists. Participant information and consent forms were translated into Simplified and Traditional Chinese by a nationally accredited translator. Chinese general practitioners, community workers and health professionals were also recruited by the BRA to participate in stakeholder interviews Data collection and analysis: Two CGHS participant focus groups (6–8 participants per group) were conducted by the BRA in Cantonese and in Mandarin, with the support of a bilingual scribe. The focus groups were audio recorded, transcribed into Simplified and Traditional Chinese and then translated into English. Transcripts, concepts and translations were checked by the BRA. Thirteen stakeholder interviews were conducted in English by the research team. The interviews were audio recorded and transcribed. Two bilingual coach reports were also obtained and analysed together with the interviews and focus groups. Transcripts were coded according to patterns in the research with research team members and BRA collectively validating the codes	Findings: Programme participants reported they formed positive relationships with bilingual coaches who provided culturally appropriate practical support. Contrary to concerns raised by stakeholders, participants were able to set goals and complete the programme. Participants also reported that GHS assisted them in increasing healthy eating and physical activity; achieving healthy weight; and improving chronic health conditions Significance and impact: Results of the study informed further refinement of CGHS and provided an evidence base for cultural adaptation of GHS into other language and cultural groups

**Table 4:** Example 4, Waterpipe smoking

Overview of the research	How we conducted the research	Outcomes
Shaping interventions to address waterpipe smoking in Arabic-speaking communities in Sydney, Australia: a qualitative study [43, 44] This study explored the perceptions and cultural meaning of waterpipe smoking in Arabic-speaking communities. Focus groups were chosen as the preferred method to understand the range of experiences and diversity of perceptions held about waterpipe smoking within a diverse community Focus groups were offered in Arabic and/or English, acknowledging the language preferences of first and second/third generation migrants Funding source: South Eastern Sydney Local Health District	Recruitment and consent: Four BRAs, recruited through existing networks, were trained in conducting and recording focus groups. Using a convenience sampling approach, focus group participants were recruited by BRAs from Arabic-speaking community groups and networks. All participants who expressed interest in participating in the focus groups were included. Participant information sheets and consent forms were translated by a nationally accredited translator into Arabic Data collection and analysis: Ten focus groups were conducted, 8 by the BRAs (Arabic and/or English) and 2 by the research team (English), and included a total of 88 participants. Two facilitators were present at each focus group. Notes were taken during the focus groups and provided to the research team. Focus groups were audio recorded where all participants agreed; participants in some groups were uncomfortable with this so only written notes were taken. The themes and subthemes from the focus groups were presented at a meeting with BRAs to validate and contextualize the key findings	Findings: Waterpipe smoking was reported to be widely practiced within the community and was related to feelings of cultural identity and belonging. The study highlighted the misconceptions that exist within communities about the health impacts of waterpipe smoking. Eleven themes were identified from the data relating to the perceptions of waterpipe smoking and possible health promotion interventions Significance and impact: This was one of the first Australian studies that explored the perceptions and cultural meaning of waterpipe smoking in Arabic-speaking communities. The findings informed a culturally responsive health promotion campaign to raise awareness of the harms of waterpipe smoking in young people from Arabic-speaking communities. The *Shisha No Thanks* project employed co-design and co-production of social media messages to address myths about the perceived relative safety of waterpipe smoking compared with cigarettes, and encouraged community conversations to challenge prevailing perceptions

Interpretative description is well suited to the approach taken in developing this framework because it avoids simple description in favour of explanations that draw on experience and contextual knowledge as interpretative filters [35]. Rigour was enhanced through describing our line of reasoning in developing the framework and describing our context and process; and ensuring our claims. Whilst we make our claims in the context in which we have undertaken this work, it is hoped that they are more broadly relevant [34].

## Results

**Fig. 1:**
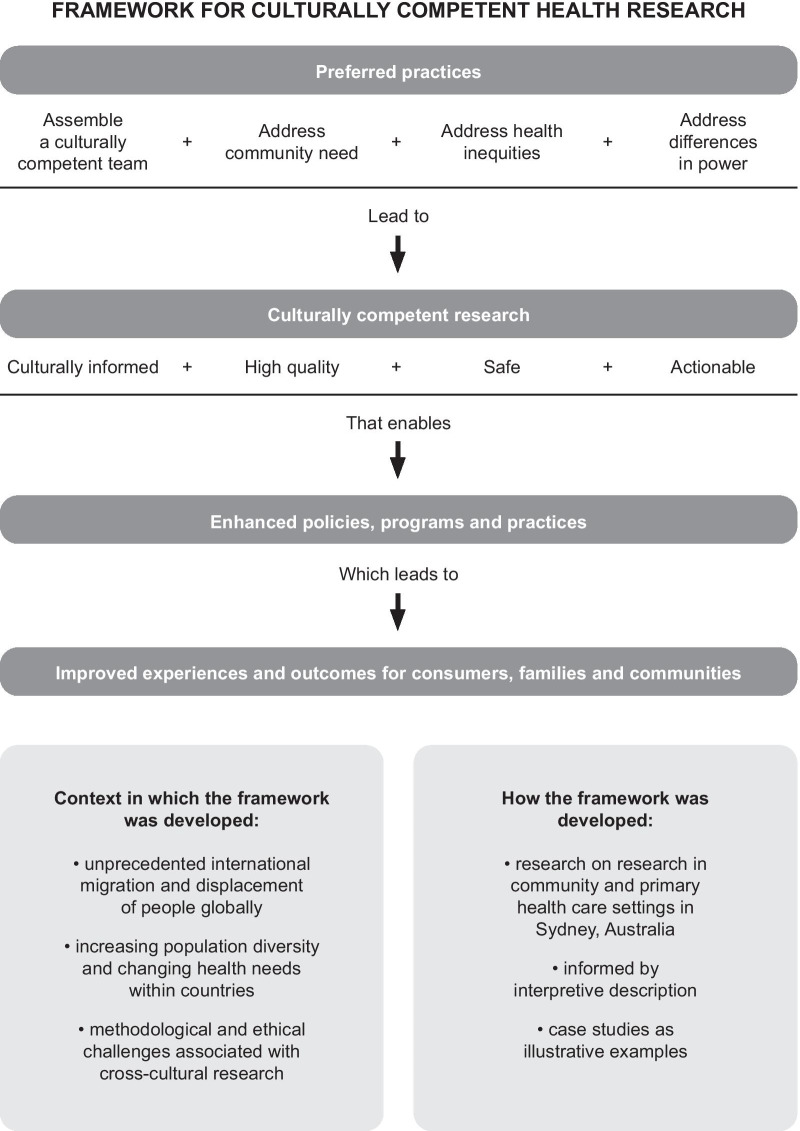
A framework for preferred practices in conducting culturally competent health research in a multicultural society.

Figure 1 presents a framework for preferred practices in conducting culturally competent health research in a multicultural society. As far as we know, it is unique in explicitly linking culturally competent research practices and outputs with evidence-based enhancements to policy and health care to deliver better health outcomes for CALD communities. Enhancements may take many forms such as targeted funding, multisector partnerships and culturally tailored services and health promotion (Tables 1, 2, 3 and 4 provide examples). The key framework elements and their interrelationships are discussed in detail below, following an explanation of how the framework was developed.

The framework is offered as a guide for targeted research with CALD communities in countries such as Australia, particularly in community and primary care settings. It is not intended to represent a gold standard or to be prescriptive as the elements may operate differently in different contexts, including research with indigenous peoples such as Aboriginal and Torres Strait Islander peoples where there are distinct needs and considerations [31].

### Elements and illustrative examples

As depicted in Fig. 1, the preferred practices include assembling a culturally competent research team and addressing health inequities, community needs, and power differentials. In Tables 1, 2, 3 and 4 (covering research overview, conduct and outcomes) we show how these practices contribute to research that is culturally informed, high quality, safe and actionable. In the integrated narrative below the key concepts are bolded.

A **culturally competent research team** (including investigators, clinician researchers and research assistants) has sufficient capability to identify the language, cultural and other barriers to research participation within the community and to ensure that, jointly, they have the right mix of skills, experience and resources to undertake the research. It is also sensitive to gender, religious and social-political issues in the target communities. In the *Fear and Shame* study [36, 37] (Table 1), the research team included clinician researchers (with the language skills and cultural background of the community) and bilingual/bicultural research assistants (BRAs). Team building included mentoring clinician researchers and training BRAs to undertake in-language data collection. In the longitudinal study of refugee children [38–40] (Table 2), the research team comprised clinician researchers supported by professional health care interpreters (HCIs). Team composition was influenced by the number of community languages spoken (10), lack of BRAs from those communities, and the preference to use HCIs for clinical assessments. The use of clinician researchers, able to respond to identified health needs and provide culturally responsive and supported referrals, enhanced** safety** for these vulnerable families. In the Chinese Get Healthy Service (GHS) [41, 42] and waterpipe smoking studies [43, 44] (Tables 3 and 4), the research teams included one or more BRAs with established community networks who were trained in recruitment, consent and conduct of focus groups. BRAs also contributed to the validation and contextualization of research findings.

Building a **culturally competent research team** strengthens research capacity within the health system (clinician researchers and HCIs) and the community (BRAs). Through mentoring and training, experienced researchers support other team members to contribute to the research being of **high quality**, that is, rigorous, transparent, reproducible and respectful of participants and the wider community. In turn, researchers benefit through enhanced understanding of the community. We recommend a community advisory group be established as part of governance arrangements and to optimize opportunity for research to be **culturally informed** and **actionable**.

**Community need** can be understood in relation to comparative need (comparisons across communities); felt need (consumer or community stated needs); normative need (defined by expert/health professional opinion); and expressed need (derived from service utilization) [45]. In our examples, we were primarily responding to comparative needs or **health inequities**, that is, differences in health status, access to health care or the distribution of health resources [46], and felt needs; although these also aligned with normative and expressed needs.

The *Fear and Shame* study evaluated an innovative applied theatre intervention to reduce barriers to accessing professional care, low levels of mental health literacy and high levels of stigma around mental illness within the Macedonian community. It followed earlier research in which community members reported a reluctance to use mental health services [37]. The Chinese GHS study evaluated the language and cultural adaptation of a mainstream programme to inform modifications to increase its accessibility and acceptability. The community had expressed an interest in participating in the programme but were reluctant to engage through HCIs with English-speaking coaches. The waterpipe smoking study responded to high waterpipe smoking rates in the Arabic-speaking community and increased risk of a range of health conditions including lung cancer, as well as the community’s request to address growing use among young people.

We acknowledge the **differences in power** between researchers and CALD communities, reflected in the ability to make and influence decisions about research questions and design as well as interpretation and dissemination of findings, and who benefits. **Safety** operates not only at an individual level but also at a community level. Within the context of this article, it refers both to the additional safeguards required for vulnerable populations (e.g. refugee children) and to building community confidence that the research will be conducted respectfully and will not be harmful. In areas in which **health inequities** exist, there is a risk that, in trying to highlight legitimate health needs, the research itself can reinforce negative stereotypes and contribute to stigma and discrimination experienced by the community. A commitment to feeding back the results of the research, and to act on the findings, further enhances community trust and **safety**.

A key feature of the illustrative examples is the enduring partnerships between researchers, health services and multicultural community organizations that have spanned multiple phases of research and research translation. In the *Fear and Shame* project, involvement of community partners included co-design and co-production of the intervention (the play was produced and performed by the Australian Macedonian Theatre of Sydney), as well as the evaluation. In the longitudinal study of refugee children, existing relationships facilitated community and service system trust and acceptance of the research, as well as uptake of supported referrals following the assessments. Feedback to the community and service system emphasized the protective factors and positive trajectories for the majority of the cohort. Community organization involvement has also facilitated co-design in the research translation phase. In the waterpipe smoking study, community members co-designed social media messages and appeared in videos and social media clips to raise awareness of the harms of waterpipe smoking.

Communities are interested in prompt responses to issues identified in research and research translation was an explicit goal in each of our illustrative examples. The findings from our studies were able to directly inform future health promotion initiatives (*Fear and Shame*; Chinese GHS and waterpipe smoking studies); health policy and health service delivery (longitudinal study of refugee children). Addressing community need, establishing trust and building community capacity to participate in the research increased the acceptability of research findings and enhanced our ability to take **action**.

## Discussion

Barriers to migrants and refugees becoming involved in health research mirror those experienced in accessing timely and appropriate health care. They include language and cultural barriers; lack of knowledge and experience with the health care system; lack of trust in government services; concerns about confidentiality and privacy (including implications for current or future visa applications for family members, especially for recent arrivals and refugees); and lack of cultural competence among research teams.

Meaningful culturally informed health research is predicated on trust and understanding on the part of the community: trust that researchers will make efforts to understand people from CALD backgrounds’ lived experiences and worldviews; trust that information will not be misinterpreted or misrepresented; and trust that the research will not harm culture itself, as has been suggested elsewhere [47, 48]. It involves ensuring harmonized, mutual benefit between communities and researchers, investing time in respectful relationships, transparency, and processes to support this [49]. Intrinsic to such research is long-term engagement with communities and commitment to community-based participatory research [50].

Research organizations, including funders, universities and ethics committees, have a critical role, because they provide the authorizing environment, resources and processes that enable culturally competent health research to occur [51]. In the field, additional time and resources are usually required to undertake research that addresses the elements of this framework. Targeted community-based research presents many challenges, and innovative approaches are often required. Practical issues for consideration include: time required to develop meaningful partnerships, defining and describing the target group (e.g. statistical indicators or self-reported identity); lack of translated and cross-culturally validated standardized measures; cost and availability of accredited HCIs and translators; and other settlement priorities (e.g. employment and education) taking precedence for communities [50, 52]. Seeding grants and small pilot studies have a place but are no substitute for sustained support to undertake long-term research across multiple sites and communities [18].

All health and medical research requires infrastructure and research capacity building. Training researchers and consumer/community representatives is particularly important. Even bilingual/bicultural researchers are at a disadvantage when it comes to their linguistic and cultural knowledge in a multicultural context. Consumers need to be inducted into the world and language of research, and supported to be involved [25]. An understanding of the framework for preferred practices will assist ethics committee members when reviewing applications.

All research takes place within broader systems of gender and sociopolitical environments [29, 30]. We do not wish to minimize the importance of the social determinants of health in producing and reproducing health inequities [53]. On the contrary, we would argue that cultural differences need to be understood alongside gender, educational status and socioeconomic status [54]. This highlights the importance of a culturally competent research team and community partners in interpreting study results and considering their implications. We are also conscious of the need to recognize the protective aspects of culture. Culture should not be problematized by health services or health researchers. Culture provides shared meaning and identity, as well as enabling mechanisms for material support. Further, culture is not static but ever-changing, as is the multicultural profile of Australian society. The status and recognition of marginalized groups within Australia has varied markedly over time [2]. Refugees constitute an especially vulnerable group given their previous exposure to human rights violations [38]. Research protocols must ensure that the rights and well-being of refugee participants are prioritized and that they are not re-traumatized through their participation.

## Conclusions

Much remains to be done to better ensure that health research meets the needs of our multicultural societies. The equitable provision of health care to the whole population requires much greater investment in inclusive and targeted research with people from diverse backgrounds. Such research is both challenging and rewarding; it is essential for evidence-based policy and programme development. This framework represents a first step towards articulating and supporting preferred practices for targeted research with CALD communities, based on the authors’ experiences in community and primary care settings in Sydney, Australia. As such it is not intended to be universally applied, although many of its aspects will have relevance for health research generally. The framework needs to be further tested and refined in different contexts.

## Data Availability

Not applicable.

## Abbreviations

BRA: Bilingual research assistant
CALD: Culturally and linguistically diverse
HCI: Health care interpreter
NHMRC: National Health and Medical Research Council
